# Unifying the odyssey: artificial intelligence for rare disease diagnosis and therapy

**DOI:** 10.1007/s12553-026-01057-y

**Published:** 2026-03-10

**Authors:** Mai-Lan Ho, Marinka Zitnik, Ronen Azachi, Sanjay Basu, Pranav Rajpurkar, Richard Sidlow

**Affiliations:** 1 Department of Radiology, University of Missouri, 1 Hospital Dr., Columbia, MO 65212, USA; 2 Department of Biomedical Informatics, Harvard Medical School, Boston, MA, USA; 3 Daliio Ltd., Tel Aviv, MS, Israel; 4 Waymark Care, San Francisco, CA, USA; 5 Department of Genetics, University of Missouri, Columbia, MO, USA

**Keywords:** Artificial intelligence, Genetic, Genome, Omics, Orphan, Rare

## Abstract

**Purpose:**

To summarize current challenges in rare disease (RD) diagnosis and therapy, highlight recent advances in artificial intelligence (AI) for RDs, and propose a model future state for RD patient care.

**Methods:**

Multidisciplinary expert-led narrative review summarizing modern practical challenges and rate-limiting steps in RD patient care, citing key clinical and research considerations with respect to regulatory and economic constraints.

**Results:**

Over 10,000 known RDs collectively affect 1 in 10 Americans, a total of over 30 million people. Annually, RDs account for over $1 trillion of annual US healthcare expenditures. Despite advances in genomic medicine, it takes 5–8 years on average to obtain an accurate diagnosis, and less than 5% of RDs currently have FDA-approved therapies. In this article, we review the history of RD diagnosis and current healthcare gaps underlying the major failures in patient care. Next, we will highlight emerging advances in genomic medicine and AI that are rapidly changing the RD landscape. Finally, we propose a target future state that integrates agentic AI for diagnosis and therapy with human-in-the-loop feedback.

**Conclusions:**

The rare disease diagnostic and therapeutic odyssey represents healthcare’s most persistent failure mode. Ongoing challenges for clinical implementation involve biological modeling, manufacturing bottlenecks, and clinical trial design. We propose strategies for artificial intelligence to restructure the traditional sequence of diagnosis-then-therapy into a proactive orchestrated system delivering personalized cures at scale.

## 1 Introduction

For the 30 million Americans living with a rare disease (RD), the journey to a diagnosis is often a multi-year “diagnostic odyssey” of clinical uncertainty, ineffective treatments, and profound frustration. This diagnostic delay, averaging 5–8 years and involving 8 or more specialists, is more than a logistical failure; it is a fundamental therapeutic barrier. With approved treatments available for less than 5% of the over 10,000 known RDs, the window for effective intervention frequently closes before a molecular cause is even identified [1]. The economic toll is staggering: RDs account for over $1 trillion in annual U.S. healthcare expenditures, with the therapeutic market alone valued at $240 billion in 2025 and projected to reach $400 billion by 2030 [2].

The RD diagnostic and therapeutic odyssey represents healthcare’s most persistent failure mode. For decades, the diagnostic and therapeutic pipelines have operated in a slow, disjointed sequence. We are now at the cusp of a paradigm shift, where artificial intelligence (AI) promises to collapse this sequence into a single, integrated engine, creating a powerful feedback loop where precise diagnosis directly informs and accelerates the creation of bespoke therapies. While this future is not yet fully realized, its core components are taking shape, offering a clear perspective on how we might finally address the long tail of human genetic disease [3].

We propose that AI can fundamentally restructure this paradigm by collapsing the traditional sequential pipeline of diagnosis-then-therapy into an orchestrated engine. This integration creates a powerful feedback loop: AI-driven population-scale screening identifies patients before irreversible damage occurs; multimodal models extract deep phenotypes that bridge the genotype–phenotype gap; and the same AI systems immediately pivot to design personalized therapies using in silico and in vitro pipelines. While AI has demonstrated remarkable capabilities in accelerating RD discovery, three non-algorithmic barriers prevent clinical deployment: biological models that fail to capture in vivo complexity, manufacturing bottlenecks that lag computational design by orders of magnitude, and absent implementation frameworks for N-of-1 clinical trials. We argue that the path forward requires not another breakthrough algorithm, but the systematic alignment of computational, biological, and economic infrastructure.

In this article, we review the history of RD diagnosis and current healthcare gaps underlying the major failures in patient care. Next, we will highlight emerging advances in genomic medicine and AI that are rapidly changing the RD landscape. Finally, we propose a target future state that integrates agentic AI for diagnosis and therapy with human-in-the-loop feedback. This orchestrated ecosystem would transform RD care from reactive, fragmented interventions to a proactive, closed-loop system delivering personalized cures at scale.

## 2 Clinical genomics: driven by cost

As genomic testing becomes more affordable, RDs are becoming increasingly reported with over 250 new monogenic disorders discovered per year [1]. At the same time, “common” disorders such as cardiovascular disease, neurodegeneration, and cancer are becoming stratified into “rare” genetic subtypes with distinct risk profiles and therapies [4]. Clinical genomic analyses are most frequently performed on blood or tissue samples, with access and payor considerations driving the choice of test. Commercial gene panels can be ordered for specific diseases or conditions, evaluating for the highest-probability causative mutations in relevant genes. If negative, patients can reflex to whole exome sequencing (WES), covering protein-coding genes and splice-site variations within 1.5% of the total genome; or whole genome sequencing (WGS), which additionally analyzes the intronic space, mitochondrial DNA, and nucleotide-repeat variations. Currently, second-generation sequencing is most commonly utilized for routine clinical care, given the rapid throughput and relatively low cost for massively parallel reading of short DNA fragments. However, the amount of data generated by WGS is enormous, with 200 gigabytes of raw data generated per human genome, and requiring ever more complex analytical methodologies. For healthcare payors to support more advanced testing, such technologies must demonstrate cost effectiveness by enabling earlier, more precise and comprehensive diagnosis that reduces downstream indirect costs [5].

## 3 Rare diseases: healthcare’s failure mode

Around 72% of all RD are thought to be genetic in etiology, of which 70% begin in childhood [6]. Several single-gene disorders have the potential to cause major morbidity and mortality in the neonatal period, so-called “devastating disorders of the newborn.” In the U.S., newborn screening (NBS) is legally mandated and evaluates for between 33 and 75 potentially treatable diseases based on state guidelines. NBS is technically limited, relying on biochemical screening of blood spots from newborn heel pricks. Furthermore, no state covers all 38 core and 26 secondary conditions listed in the Recommended Uniform Screening Panel (RUSP) from the U.S. Department of Health and Human Services (HHS) [7]. Many centers are starting to adopt rapid whole exome or genome sequencing (rWES, rWGS) for broader disease coverage using the same blood spot cards. However, due to the need for quick actionable decision-making in the neonatal ICU, rapid analysis algorithms currently prioritize turn-around time (hours) over depth of information, flagging only high-probability conditions in acutely ill patients. Agnostic use of rapid newborn sequencing is currently not performed, due to ethical issues regarding variants of unknown significance (VUS) that are identified incidentally, preclinically, and/or linked to diseases without known treatments [8].

In older children and adults, RD screening is dependent on primary care physicians, who already struggle with systemic workforce shortages and limited resources. Clinically, RD patients can present with nonspecific features, such as developmental delay and abnormal facies, that are subjective and difficult to distinguish from common “bread and butter” disorders. Without a high index of suspicion for RD, patients with multisystem disorders may be overlooked or referred to many different specialists who lack RD domain expertise, resulting in “false leads” with further confusion and delays in care. At the population level, RDs create disproportionate burdens on healthcare systems with excessive ordering of costly tests and treatments that fail to help patients [9].

## 4 Genomic pitfalls and investigational gaps

WGS results can vary greatly between laboratories due to variable quality and depth of sequencing, differing annotation databases for meaningful genetic variants, and parental genomic information. If the raw data is available, genomic scientists may elect to reprocess it using different or updated bioinformatic algorithms and databases. Continual literature reanalyses are crucial, since a single case report can result in a VUS being reclassified as pathogenic. Functional genomics data is also highly fragmented and variable in quality, requiring better access and standardization to accelerate clinical uptake. Furthermore, a negative clinical WGS does not exclude the presence of genetic disease. Current short-read techniques can miss repeat expansions, as seen in Huntington disease and Fragile X syndrome; and large chromosomal aberrations [10]. Though more expensive, third-generation (long-read) sequencing is preferred for characterizing complex genomic regions; is sensitive to single-base modifications such as native DNA methylation; and enables more accurate haplotype phasing for disease risk and inheritance patterns [11].

Multi-omic testing is still investigational and not covered by clinical payors, but can offer improved understanding of disease mechanisms by revealing interactions between different biological levels. The emerging fields of epigenomics, transcriptomics, proteomics, metabolomics, and metagenomics can provide complementary information regarding gene expression, transcription, translation, stability, and interactions. For example, DNA methylation and histone modification play major roles in cancer and inflammation. RNA spliceopathies are responsible for retinitis pigmentosa, myelodysplastic syndromes, and muscular dystrophies. Proteinopathies are implicated in the majority of neurodegenerative disorders. Dynamic interactions between the host genome, microbiome, and exposome continually influence the state of health and disease [12].

## 5 Clinical pitfalls in RD evaluation

Clinical evaluation of RDs is notoriously difficult with nonspecific history, subtle physical exam findings, and unique domain expertise required to order and interpret the appropriate diagnostic tests. Additional confusion can arise due to complex heritability and/or weak effect sizes with multiple pathway interactions, epigenetic, and environmental factors. Common genetic phenomena include variable penetrance (likelihood of expressing a visible phenotype), expressivity (degree of phenotypic expression), latent phenotypes (hidden but unmasked by certain conditions), blended phenotypes (mixed due to incomplete dominance), polygenic inheritance (multiple genes contributing to a single trait), and/or pleiotropy (single gene influencing multiple unrelated traits). Often, difficult cases require multidisciplinary expert teams to come together and identify unique combinations of subtle phenotypic alterations across multiple domains. Ongoing work in the field involves “geno–pheno correlation”: relating genotype to phenotype via vertical integration of information from micro- to macroscale [13–18].

When the evidence is inconclusive, medical geneticists must make an informed decision based on multi-domain knowledge of genetic, embryologic, and developmental mechanisms, detailed phenotypic batteries, and ethical implications for patients and their families. The concept of endophenotype or intermediate phenotype, a heritable biomarker associated with a particular disease or trait, can help bridge the geno–pheno gap. Endophenotypes (intermediate phenotypes) reflect mesoscale processes that are related to genetic mechanisms, yet are more readily measurable than standard clinical phenotypes. Examples include quantitative risk scores based on electronic health records (EHR), laboratory tests, or medical imaging [19, 20]. Deep phenotyping is a broader concept that involves synthesizing advanced information across multiple dimensions, including clinical, molecular, and behavioral, to better characterize the complexities and nuances of individual phenotypes [21, 22].

## 6 Healthcare data and workforce limitations

To capture RD patients early, primary care physicians need to maintain a high index of suspicion with a low threshold for genetic consultation. However, it is both unrealistic and cost-prohibitive to refer every atypical primary care patient to a geneticist. In light of widespread human workforce shortages, there is a critical need for large-scale population screening of RD using automated, objective, and high-throughput technologies. Unfortunately, data for RD is highly fragmented, with variable-quality information in multiple domains disseminated across space and time. There are no systemic data warehouses for RD analysis, and data pooling is unrealistic given that each individual’s information resides in multiple different hospitals and laboratories. Various attempts are being made to consolidate multicenter data by federal, academic, and private organizations as well as patient foundations. However, progress in data sharing is greatly hindered by privacy, ethics, and cost considerations across the vast landscape of RDs. Even bioinformatic annotation and analysis methods vary widely, with no standardized international syntaxes for consistent data exchange [23, 24].

Recent AI innovations, particularly generative AI, can help overcome data and workforce challenges with powerful foundation models discovering novel patterns within unstructured multimodal EHR and genomic data. Although AI cannot replace human experts, it can help integrate, annotate, and update multicenter datasets to accelerate research translation, mechanistic understanding, and expert review of high-probability cases. In addition, AI can help provide standardized and quantitative metrics to augment human expertise including clinical risk scores, endophenotype discovery, and composite phenotyping based on multimodal data (clinical notes, laboratory values, audio/video recordings, medical images, genomic sequences) [25–27].

In light of these exciting technological advances, it is important to concurrently address the implementation challenges of AI screening in the EHR. Primary care physicians already suffer from “alert fatigue” with repeated exposure to low-value and false-positive notifications, leading to desensitization with delayed or inadequate responses to critical warnings. Therefore, the ultimate success of AI for RD clinical support will hinge on the ability to provide high-yield, low-volume alerts that are seamlessly integrated into the daily workflow [28].

## 7 RD market: growing yet constrained

The RD diagnostics market is valued at $1.5 billion in 2026 and estimated to reach $8 billion by 2030, for a compound annual growth rate (CAGR) of 14.23%. This includes startups and established companies, government consortia, academic research groups, and patient foundations. Patient-directed services include symptom matching, networking, education, and connections to clinical trials, centers of excellence, and physician specialists based on disease. Many companies are analyzing EHR data using various AI approaches (machine learning, deep learning, natural language processing, large language models) to accelerate identification of potential RDs from structured and unstructured medical records. Federal and academic research groups maintain a number of publicly available omics databases, atlases, bioinformatic models, and literature review tools. Genomic laboratories have applied AI to various use cases including faster base calling, improved variant interpretation, error correction, and omics data integration. AI tools are also available to analyze facial morphometry, audio/video recordings, ECG, EEG, fundoscopy, skin analysis, radiology, and pathology data [29]. Progress has been limited by insufficient clinical expert feedback, highlighting the need for humans-in-the loop to achieve successful model training, evaluation, and deployment [26].

The RD treatment market is valued at $240 billion in 2026 and estimated to reach $400 billion by 2030, for a CAGR of 11.93%. Several companies are leveraging AI for drug discovery and repurposing, protein structure and binding sites, and clinical trials optimization [30]. The Orphan Drug Act of 1983 and additional legislation have incentivized pharmaceutical development for RDs [31, 32]. Over the past five years, 143 drugs launched with orphan drug designation in the U.S., representing over half of new U.S. Food and Drug Administration (FDA) active substance approvals [33]. A high unmet need remains, with 95% of over 10,000 RDs lacking available treatments. Even when promising therapeutic candidates are developed, pharma companies experience major challenges enrolling RD patients early enough to show treatment efficacy. This situation further emphasizes the need to accelerate RD diagnosis and route candidates for appropriate clinical trials [1].

## 8 AI for RD diagnosis

### 8.1 From fragmentation to multimodal integration

The foundational challenge in RDs has always been the lack of information, both in terms of availability and quality standards. The diagnostic odyssey is a symptom of a healthcare system struggling to recognize faint signals within noisy and unstructured clinical data. Diverse AI algorithms are being applied for RD diagnosis by government, academia, industry, and foundations, unfortunately without a coherent focus or direction. Progress is hindered by the lack of an open and interoperable ecosystem for data sharing and collaboration across multiple diseases, sectors, and populations [26].

With decreasing costs of WGS, massive amounts of patient genomic data are becoming widely available. However, the greater challenge lies in functional interpretation—linking a variant of unknown significance (VUS) to a subtle constellation of clinical phenotypes scattered across unstructured EHRs, imaging reports, and specialist notes. This geno–pheno gap represents the central chasm in RD care: a space where human cognition struggles to synthesize disparate, longitudinal data points into a coherent diagnosis [5].

Though currently limited for RD applications, generative AI (GAI) may help address longstanding challenges with scarce, unstructured, and low-quality data in the field. Multiple groups have demonstrated the feasibility of pretrained foundation models to improve detection of common diseases. In addition, generalist models can be fine-tuned on RD datasets in order to extract unique phenotypic signatures [34]. Large language models (LLMs) tested on diverse retrospective datasets show the potential to augment RD diagnosis through conversational patient engagement, improved physician ontologies, and population EHR screening [35–37]. Vision language models (VLMs) are also being used to analyze image, video, and audio data in general medicine and subspecialties such as radiology, pathology, ophthalmology, and surgery [38–41].

Despite these promising preliminary results, prospective GAI deployment in the clinical setting requires expert human oversight at critical decision points to provide real-world feedback; define appropriate performance metrics; and mitigate failure modes including hallucination and bias. For example, LLMs struggle with complex patient cases that involve multi-step reasoning or long patient trajectories. Genomic data is also sparse and less structured in essential content compared to human language, so LLM tokenization may yield inaccurate and nonsensical results [42, 43].

In the long run, GAI algorithms trained on multimodal RD data have the potential to detect unique patterns systematically across thousands of patients. An innovative primary care paradigm would leverage AI to proactively screen entire health system populations as a first-line approach. Foundation models could be trained to sift through years of unstructured multimodal data—visit notes, laboratory values, electrophysiology recordings, and medical images—to discover new quantitative biomarkers of RDs. By integrating multiple historically siloed data sources, GAI models would construct holistic deep phenotypes to bridge the geno–pheno gap with unprecedented fidelity [44, 45].

## 8.2 Cost-effective screening

**Fig. 1:**
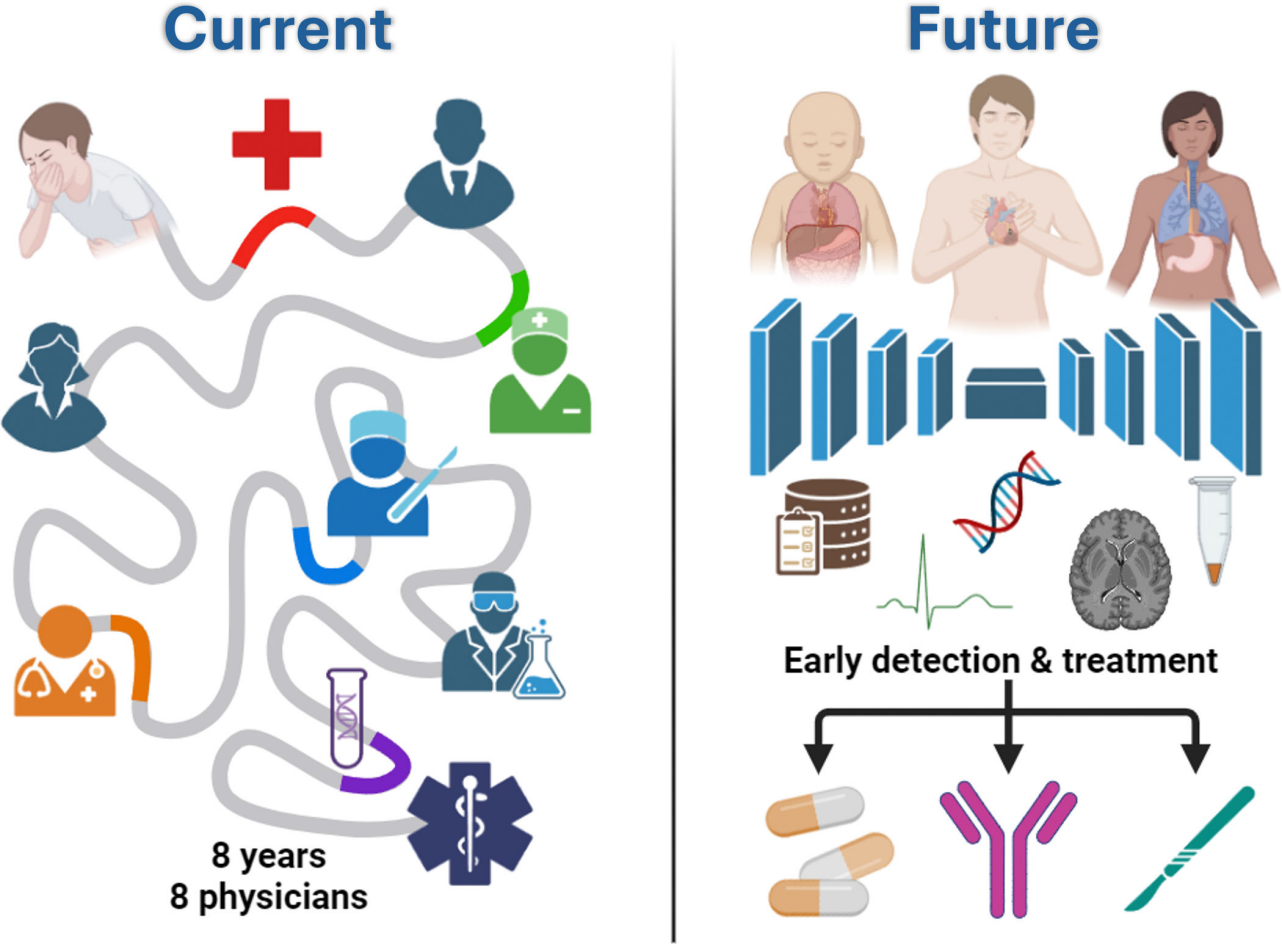
The diagnostic odyssey transformation. ***Current*:** Traditional pathway for rare diseases with multiple specialists, dead ends, and delayed diagnoses that often preclude effective treatment. ***Future*:** Proactive individualized AI screening of multimodal patient data, enabling early detection and initiation of appropriate therapies. Created in BioRender.com

As modern healthcare costs skyrocket, it becomes essential to optimize cost-effective approaches to RDs. In particular, indiscriminate genomic sequencing should be avoided, as it becomes prohibitively expensive to scale and generates massive amounts of data with negligible clinical value. Furthermore, the results are challenging to analyze and can elevate psychological anxiety, while also risking false-positive diagnoses and unnecessary treatments. A cost-effective paradigm for RD care should leverage the highest-value technologies to identify at-risk patients early, selectively apply high-probability tests, deliver effective therapies, and minimize downstream costs. If designed correctly, these workflows would cost a fraction of the current $1 trillion annual health care expenditures for RDs, enabling early detection and treatment with minimal downstream costs (Fig. 1).

Since the majority of RDs are pediatric-onset and potentially devastating if undiagnosed, young children should be screened early in life: at birth, or even prenatally for high-risk pregnancies (using maternal cell-free DNA). As genomic testing becomes cheaper, rapid WGS should supplant NBS for broader and more consistent disease screening in newborns. Furthermore, WGS specimens and raw data can be biobanked for future reference and potential reanalysis using more comprehensive algorithms, should the patient develop symptoms later in life [8].

For older children and adults, AI screening of available EHR data should be the first step to identify and recommend high-risk individuals for genetic evaluation. AI can generate objective and reproducible thresholds for population screening using basic medical information reported by patients and/or general practitioners. This process will lift the burden from primary care physicians and ensure that limited genetics resources are directed toward the highest-probability patients. Low-access areas can be supported by telemedicine programs using smartphones and/or wearables to enable remote phenotyping of facial morphology, body proportions, voice characteristics, eye tracking, and movement patterns. AI can also augment information extraction and provide unique insights into disease mechanisms, such as new symptom clusters or subtle features that deviate from population norms [46].

AI can also help genomicists interrogate variations in data quality and bioinformatic approaches. To date, researchers have focused on computational extremes to identify genetic loci: linkage analyses to identify heritable regions with large effect sizes, and genome-wide association studies to detect population-level associations between common variants and traits. There is a critical need for standardized and publicly available omics toolkits that can perform rapid “one-stop-shop” quality assessment, genomic annotation, molecular modeling, variant curation, phenotype prediction, and personalized treatment [10, 11]. GAI agents are ideally suited for this purpose, as they can continually retrieve and learn from multiple data sources including research literature, public databases, clinical trials, and expert reviews [34].

For the most complex unsolved cases, advanced “4D multi-omic” testing may be necessary, collecting spatial, temporal, and molecular data across multiple biological scales, with AI essential for integrating these massive datasets into actionable insights. This would collect relevant spatial, temporal, and molecular data, for example the emerging fields of interactomics (total set of interactions among molecules) and exposomics (total lifetime environmental exposures) [12]. The key objective is to find patients before the critical point at which irreversible organ damage occurs, thereby closing the therapeutic window. By moving from a reactive symptom hunt to a proactive, population-scale screening process, AI can generate a stream of accurately diagnosed patients who are ready for the next step: treatment.

## 9 AI for RD treatment

### 9.1 Many barriers, few successes

Over the past decades, the Orphan Drug Act and FDA special designations for unmet medical needs have spurred the pharmaceutical industry’s interest in developing RD therapies [31–33]. However, current initiatives remain insufficient to address increasing patient needs. Bringing a pharmaceutical to market averages 10–15 years and over $1 billion of investments in research & development, clinical trial, regulatory, and manufacturing costs [47]. Furthermore, around 90% of new drugs fail in clinical trials, with half due to human toxicities that were not predicted by preclinical studies [48].

To facilitate bench-to-bedside translation, the FDA recently established the plausible mechanism pathway to approve personalized RD therapies, for which traditional clinical trials are nearly impossible. This allows for conditional approval based on a small number of patients with strong biological evidence and meaningful clinical improvement [49]. Furthermore, both the FDA [50] and National Institutes of Health (NIH) [51] have announced prioritization of human-based research technologies and reduction of animal experimentation, based on cost, ethics, and accuracy concerns [52].

Attempts to accelerate drug development pipelines should be coupled with strong governance frameworks to ensure that AI systems are deployed ethically, responsibly, and fairly. Many controversies around RDs are attributed to poor understanding and ineffective therapies, resulting in insurance/employment discrimination and social stigmatization. For example, polygenic risk scores estimate the likelihood of developing certain diseases relative to the population, but can influence providers to overdiagnose preclinical diseases and overtreat secondary findings. At the other extreme, positive testing for delayed-onset and intractable conditions (such as Huntington disease or amyotrophic lateral sclerosis) can yield severe psychological distress with negligible likelihood of cure [53].

### 9.2 Intelligent therapeutic design

The ultimate potential of AI in RDs lies in coupling the aforementioned diagnostic engine to a novel therapeutic development workflow. The current linear progression of molecular target discovery, preclinical animal models, and large-scale human clinical trials is ill-suited to ultrarare and nanorare disorders affecting fewer than 30–100 patients worldwide. Rather, the future of AI-designed therapeutics lies in a rapid, iterative, and closed-loop system based on patient-specific biology. After RD diagnostic resolution, therapy begins by identifying the biological target (causative molecular defect) and aligning it with an appropriate therapeutic modality. For drug design, target properties define the route of administration. Loss-of-function targets with preserved reading frame and accessible tissues point to gene augmentation or mRNA therapy. Gain-of-function or dominant-negative targets prioritize allele-selective silencing, RNA interference, or editing. Splice defects can be treated with antisense oligonucleotides. Extracellular or secreted proteins favor antibodies or protein therapeutics. Enzyme deficiencies suggest replacement or chaperones. Cell loss or circuit dysfunction motivates cell therapy approaches. Network-level dysregulation with multiple interrelated effects often suits small molecules or rational combinations [54].

With the target and modality selected, drug design and optimization become a search over a shared intervention space. AI can make pharmaceutical routing data-driven; we envision that multimodal AI learners will fuse genomics, proteomics, imaging, and clinical trajectories to infer target class, quantify druggability, and anticipate delivery constraints such as tissue access or immune context. Structure-aware and atom-scale AI models can evaluate whether a particular molecular feature pocket or splice pattern is amenable to a given modality [55]. Knowledge-graph reasoning aligns the target with pathways and compensatory circuits [56]. Drug repurposing [57] and *de novo* design [58] use patient-conditioned priors and scoring functions. Retrieval-augmented and agentic systems surface similar cases, mechanistic evidence, and preclinical results to match the target and modality [59]. Generative [60, 61] and quantum computing [62] models can propose sequence edits, chemotypes, delivery vectors, or protein scaffolds consistent with biophysical and exposure constraints. Combinatorial optimizers explore multi-drug regimens and drug-plus-novel-compound strategies under interaction and exposure limits and simulate dose and schedule [63]. Multi-objective optimization trades off benefit, risk, and time to treatment, producing a ranked slate of drugs or drug candidates rather than a single option [64]. Each AI-generated drug proposal carries a structured rationale, calibrated uncertainty, and counterfactuals that reveal which observations or design changes would shift rank. This target-to-modality decision can fix the intervention design space and hand off a target-conditioned plan to candidate generation. A policy-aware model can additionally integrate benefit, safety, feasibility, and time to treatment and recommend hybrid strategies, for example an existing drug paired with a novel compound in order to balance efficacy and safety [65].

**Fig. 2:**
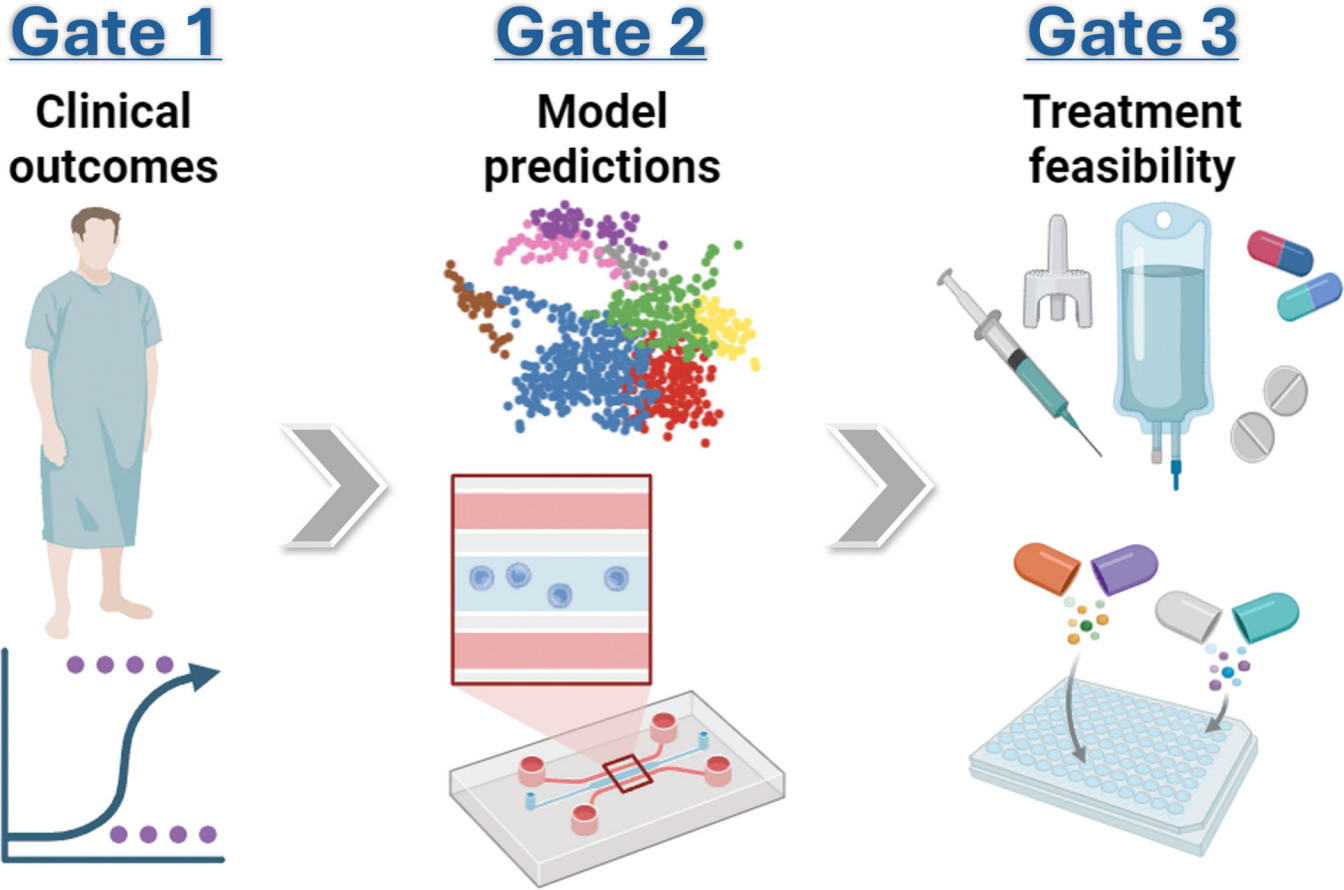
Decision gates for therapeutic development. ***Gate 1*** evaluates anticipated patient benefit based on clinically meaningful outcomes. ***Gate 2*** tests concordance with patient-derived in vitro or in silico models. ***Gate 3*** verifies treatment feasibility in context including dose, route, and interactions. Created in BioRender.com

To address current challenges in RD therapeutics research, we propose that progression from in silico proposals to treatment should pass through key decision gates that prioritize practical utility. ***Gate 1*** evaluates expected net benefit at the patient level, anchored to minimal clinically important differences for outcomes that matter. ***Gate 2*** tests concordance with human-derived biologic models such as induced cells, organoids, or engineered tissues using a battery that integrates molecular, morphological, and functional readouts. In the future, AI can align digital twin/avatar responses with patient features to estimate transport from bench to bedside and minimize the need for animal testing [66]. ***Gate 3*** verifies efficacy and safety margins in context, including dose and route feasibility, interaction risk for combinations, and operational readiness in the clinical setting. Drug candidates that clear the gates will move to treat-now pathways. Candidates that do not clear a gate require additional targeted experiments to optimize pathways or design iterations to lift the limiting criteria. This approach will enable continual recalibration, rapid escalation when net benefit exceeds threshold, and de-escalation when validity erodes (Fig. 2).

### 9.3 Real-world bottlenecks

The powerful vision for integrated RD theranostics is presently impeded by three stubborn chokepoints [23, 67, 68]. First, current biological models, both in vitro and in silico, still miss too much real-world complexity. The most advanced “virtual cell” models couple metabolism and signaling pathways, but do not include emergent higher-order features such as the organization of phase-separated compartments or the dynamics of mitochondrial heteroplasmy, leading to fragile dose-response predictions [69]. In the lab, patient-derived organoids fail to capture the multi-system crosstalk that critically impacts drug response, including cell maturation, extracellular matrix, and integrated neural, vascular, and hematopoietic networks [70]. In light of this fidelity gap, many AI-nominated hits that succeed in theory will continue to fail when they enter the complex environment of a living organism. Until synthetic models can better recapitulate the full spectrum of biologic complexity from nano- to macroscale, AI search engines will keep reporting answers that biology later vetoes.

Second, the throughput of Good Manufacturing Practices (GMP)-compliant facilities currently constrains the treatment calendar. Although an AI algorithm can design a personalized ASO or adeno-associated virus (AAV) vector in hours, actual physical production of a clinical-grade batch may take months or quarters. Individualized therapies must pass through the contract development and manufacturing (CDMO) sector, which is already running short on skilled staff and clean-room slots. The market for therapeutic oligonucleotides is projected to triple by 2029, a growth rate that far outpaces facility expansion. There is a frustrating disconnect with the discovery process being orders of magnitude faster than the production process, resulting in delayed access to potentially life-saving treatments. In future, AI could help address these physical limitations by enabling robotic automation, integrated process monitoring, predictive maintenance, formulation optimization, quality control, supply chain management, patient distribution, and regulatory intelligence [71].

**Fig. 3:**
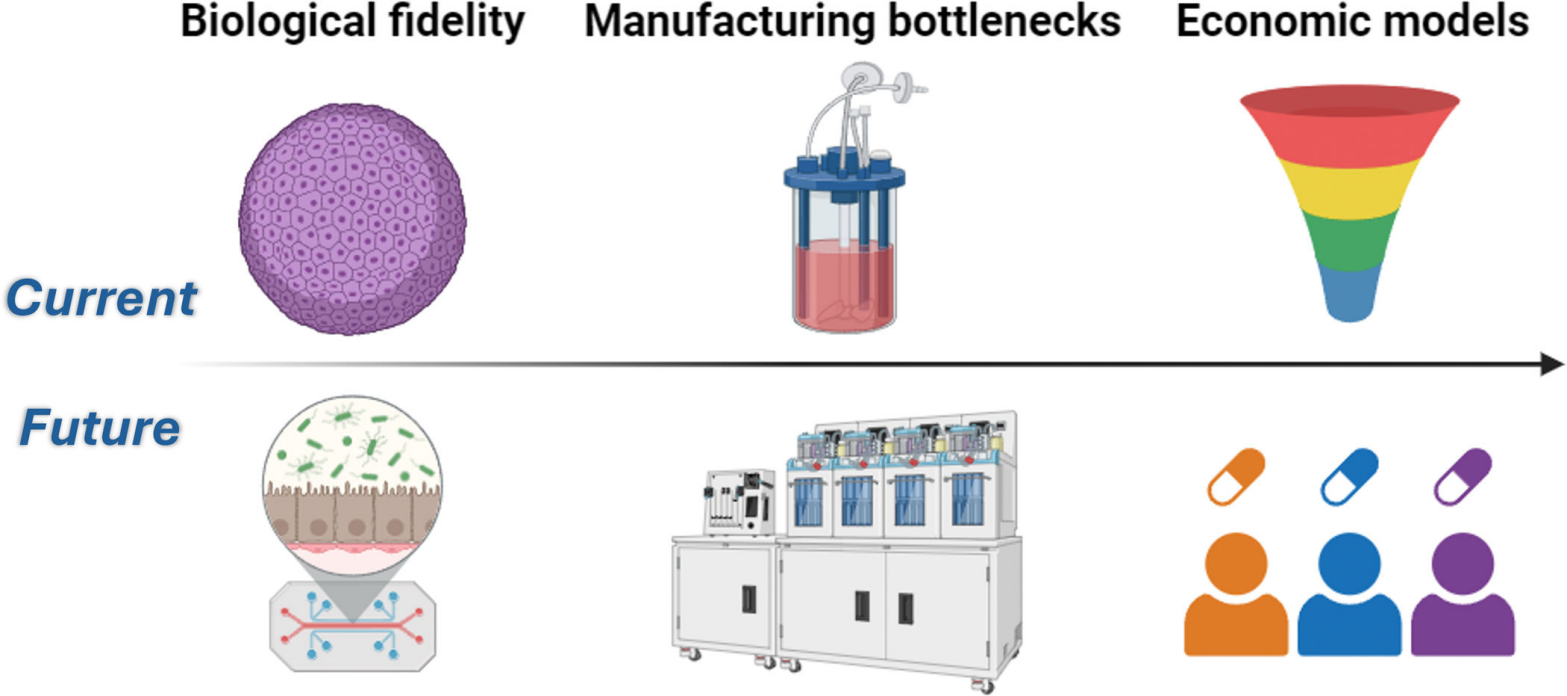
Real-world chokepoints that can be addressed using AI. ***Biological fidelity*:** Laboratory organoids currently lack biological complexity, requiring improved multisystem modeling for real-world fidelity. ***Manufacturing bottlenecks*:** Facilities limited by clinical batch production can be accelerated using robotic automation with continuous supply chain management. ***Economic models*:** Reimbursement policies favor common medications with tiered formularies, whereas rare diseases necessitate commercialization of N-of-1 frameworks. Created in BioRender.com

Third, the economic model for RD therapies remains unsolved. The question of who pays for expensive and personalized therapies in this ultra-niche market is a critical barrier to widespread adoption. Philanthropic models like the n-Lorem Foundation have been pioneers, treating dozens of “nano-rare” patients with personalized ASO technologies and proving the technical feasibility of the N-of-1 approach [72]. However, charity alone cannot support the hundreds already approved for help, let al.one the future demands. AI may make the discovery of a cure astonishingly cheap, but if the final product remains prohibitively expensive and unreimbursed, it will remain inaccessible to patients. Therefore, close cooperation between government, foundation, and industry partners is sorely needed to create a sustainable infrastructure for research, development, commercialization, and distribution of RD technologies. Until actuarial approaches and reimbursement policies are established for therapies that cannot achieve the statistical power of a traditional clinical trial, mainstream health systems and payors cannot budget for bespoke treatments [73] (Fig. 3).

## 10 AI orchestration: end-to-end care

Within the booming RD market, numerous research and commercial AI tools are emerging for each stage of care: population screening and deep phenotyping, genomic interpretation and integrated diagnosis, target identification and therapy design, model validation and iteration, manufacturing and deployment. These initial prototypes can be further developed into autonomous AI agents that are fine-tuned to specific domains, perform complex multi-step reasoning, update knowledge from database/literature searches, and dynamically learn from feedback. The final layer involves an AI orchestrator agent that connects modular domain-specific agents and integrates expert human supervision at critical decision points. This multi-agent system can be utilized to manage the RD diagnostic and therapeutic odyssey from end-to-end, including reinforcement learning, experimentation, and collaborative decision making [74–77]. Orchestrated AI represents the ideal paradigm for RD care: seamless connection of evolving data, research, and testing capabilities to find a personalized cure for each individual [78].

## 11 Conclusion

Despite advances in genomic technologies, our current healthcare system is fragmented and fails to correctly diagnose and treat RD patients in a timely manner. AI shows transformative potential in accelerating RD diagnosis and treatment, yet significant implementation challenges remain. The path forward requires a focused effort on the longstanding barriers that constrain deployment: improving the biological fidelity of models, scaling distributed and modular manufacturing, and developing novel payor reimbursement frameworks. The future of RD care will not be delivered by a single breakthrough algorithm, but by the systematic orchestration of these computational, biological, and economic solutions. By building this unified ecosystem, we can transition from celebrating one-off miracles to delivering personalized medicine as the standard of care.
